# Heterologous ORFV–Ad26 vaccination broadens antibody breadth and amplifies cellular immunity against SARS-CoV-2 spike

**DOI:** 10.3389/fimmu.2025.1715442

**Published:** 2025-11-18

**Authors:** Alena Reguzova, Verena Haug, Melanie Müller, Madeleine Fandrich, Alex Dulovic, Ralf Amann

**Affiliations:** 1 Institute of Immunology, University Hospital Tübingen, Tübingen, Germany; 2 Institute for Tropical Medicine, Travel Medicine, and Human Parasitology, University Hospital Tübingen, Tübingen, Germany; 3 NMI Natural and Medical Sciences Institute at the University of Tübingen, Reutlingen, Germany

**Keywords:** Orf virus, viral vector, vaccines, heterologous prime-boost, SARS-CoV-2, Jcovden

## Abstract

The durability of vaccine-induced immunity is often limited by waning responses, antigenic drift, and anti-vector immunity, highlighting the need for innovative vaccination strategies. Heterologous prime–boost approaches can help overcome these barriers by exploiting the complementary strengths of distinct platforms. Here, we evaluated a replication-deficient Orf virus-based spike vaccine (ORFV-S) in combination with the licensed Ad26 vector vaccine Jcovden (Ad26.COV2.S), using SARS-CoV-2 model. In a clinically aligned intramuscular immunogenicity screen in mice, Jcovden induced strong early anti-spike antibody responses but showed limited boostability, whereas ORFV-S was highly boost-responsive. Mixed regimens outperformed both homologous schedules. ORFV-S prime followed by Jcovden boost elicited the highest spike-binding antibody titers and vigorous CD4^+^ and CD8^+^ T-cell responses with a dominant Th1 profile. In the reverse regimen, ORFV-S boost improved inhibition across multiple SARS-CoV-2 variants, including immune-evasive strains and indicated qualitative improvements in the response breadth. Together, these findings suggest that sequence-dependent effects allow heterologous schedules to emphasize either cellular or humoral arms of the adaptive response.

## 1 Introduction

While many licensed vaccines have proven effective in controlling infectious diseases, their long-term efficacy is often compromised by waning immunity and the ongoing evolution of viral pathogens (1–4). The emergence of immune-evasive variants poses a persistent challenge to the durability and breadth of vaccine-induced protection (2, 3, 5). This problem is particularly evident in rapidly mutating viruses such as SARS-CoV-2 (4, 6), influenza (7, 8), and HIV (9, 10), where antigenic drift and immune escape can significantly reduce vaccine effectiveness and lead to breakthrough infections. As a consequence, regular booster vaccinations have become necessary, yet homologous boosting can be hampered by anti-vector immunity and diminishing immunogenicity. These challenges underscore the need for adaptable vaccination strategies that elicit robust, broad, and long-lasting immune responses against a wide range of viral threats (11–13).

Heterologous prime–boost vaccination, in which distinct vaccine platforms are administered sequentially, has emerged as a compelling strategy to overcome limitations associated with homologous regimens and to enhance immunogenicity (14). By combining vectors that differ in antigen delivery and innate-sensing profiles, such approaches can bypass vector-specific immunity, reduce immune tolerance, and amplify the magnitude and breadth of humoral and cellular immune responses (15, 16). During the COVID-19 pandemic, schedules pairing an adenoviral prime with an mRNA boost consistently outperformed homologous regimens in clinical and preclinical settings (17–21). Comparable benefits have been reported across other indications, including Ebola, tuberculosis, malaria, and HIV, where combinations of distinct viral vectors elicited stronger and more durable immune responses than single-platform strategies (22–26). Together, these findings demonstrate that heterologous vaccine combinations can systematically enhance vaccine efficacy across a broad range of pathogens.

Jcovden (Ad26.COV2.S), developed by Janssen, is a recombinant, replication-incompetent adenovirus serotype 26 (Ad26) vector vaccine that encodes a prefusion-stabilized spike protein (27). Early phase 1/2 studies explored a two-dose schedule, yet the second homologous dose boosted neutralising-antibody titres only very modestly (28). This increment considered clinically marginal when weighed against increased reactogenicity and manufacturing demand and therefore, regulatory authorities authorised Jcovden as a single-shot vaccine (29).

Beyond COVID-19, the Ad26 platform has induced durable immune responses in candidate vaccines against Ebola virus (30), HIV (31), and Zika virus (32). Nonetheless, anti-vector immunity remains a critical bottleneck. Pre-existing or vaccine-induced antibodies directed against the Ad26 capsid can curtail transgene expression and dampen recall responses, thereby limiting the incremental benefit of homologous booster doses (33, 34). Pairing Ad26 with a distinct vector therefore represents a rational strategy to bypass vector-specific immunity and further enhance vaccine potency (35).

Orf virus (ORFV), a member of the *Parapoxvirus* genus, combines several attributes that make it an attractive vector platform for vaccine development: (i) its natural host-range restriction and genetic attenuation confer excellent safety (36–38); (ii) its large double-stranded DNA genome allows accommodation of multiple transgenes under independent viral promoters (39), and (iii) the virus induces only transient and low-level anti-vector immunity, permitting repeated administration without loss of efficacy (37, 40, 41). Moreover, ORFV-based vectors have been shown to trigger potent innate sensing via cGAS–STING which translates into strong humoral and cellular immune responses after vaccination (42, 43). As a proof of concept, the multi-antigenic candidate Prime-2-CoV, co-expressing prefusion-stabilised SARS-CoV-2 spike and nucleocapsid proteins, has demonstrated robust immunogenicity in hamsters and non-human primates (41), absence of systemic replication *in vivo* (38), and an excellent safety profile in a first-in-human phase I trial (44, 45).

Given the advantageous features of ORFV and the well-established potency of the Ad26 vector (Jcovden), we hypothesized that combining both platforms in a heterologous prime–boost regimen might elicit synergistic immune responses. To specifically investigate this hypothesis, we employed a streamlined ORFV vector encoding only the spike antigen (ORFV-S) rather than the previously characterized multi-antigenic Prime-2-CoV. Using SARS-CoV-2 as a clinically relevant and immunologically well-characterized model, we compared heterologous strategy with homologous two-dose regimens in which each vaccine was administered alone. Our findings demonstrate that this heterologous approach significantly improves key immunological parameters, thereby providing a rationale for broader applications of mixed-platform strategies to enhance vaccine efficacy against diverse infectious diseases.

## 2 Materials and methods

### 2.1 Ethics statement

Peripheral blood mononuclear cells (PBMCs), subsequently used for monocyte isolation, were obtained from buffy coats provided by the University Hospital Tübingen, Center for Clinical Transfusion Medicine. The use of human biomaterials was approved by the Ethics Committee of the Medical Faculty of Eberhard Karls University Tübingen and the University Hospital Tübingen (project number 507/2017B01).

Animal housing and experimental procedures were carried out in accordance with the recommendations of the Federation of European Laboratory Animal Science Associations (FELASA) and the applicable regulations of the regional authorities. All animal experiments were performed by Synovo GmbH, Tübingen, Germany. The study protocol, including all procedures such as surgery, anesthesia, and euthanasia where applicable, was approved by the regional authority under project license no. 35/9185.81-7/SYN 12/20.

### 2.2 *In vitro* transgene expression

To assess antigen expression mediated by the ORFV-based vaccine vector encoding the SARS-CoV-2 spike protein (ORFV-S) and by Jcovden (Ad26.COV2.S), flow cytometry was performed in HEK293-derived VPC 2.0 cells and human CD14^+^ monocytes, while immunofluorescence microscopy was performed in CD14^+^ monocytes only.

HEK293-derived VPC 2.0 cells (cat. no. A49784, Thermo Fisher Scientific, Waltham, MA, USA) were seeded in 24-well plates (Greiner Bio-One, Frickenhausen, Germany) at a density of 5 × 10^5^ cells per well in 500 µL Virus Production Medium (cat. no. A4817901, Thermo Fisher Scientific). Peripheral blood mononuclear cells (PBMCs) were isolated from buffy coats by Ficoll density gradient centrifugation (Biocoll Separation Solution, Merck KGaA, Darmstadt, Germany). CD14^+^ monocytes were subsequently purified using magnetic cell sorting with CD14 microbeads (MACS, Miltenyi Biotec, Bergisch Gladbach, Germany) and seeded in 96-well plates at a density of 1 × 10^5^ cells per well in 200 µL Iscove’s Modified Dulbecco’s Medium (IMDM; Lonza, Köln, Germany) supplemented with 10% heat-inactivated FCS (Sigma-Aldrich, St. Louis, MO, USA). Cells were infected with ORFV-S at a multiplicity of infection (MOI) of 0.3–30 or with Jcovden at an MOI of 0.3–100, or left uninfected as controls. Sixteen hours post-infection, cells were harvested for analysis. Viability was assessed using Zombie Aqua Fixable Viability Dye (cat. no. 423102, BioLegend, San Diego, CA, USA). For surface staining, cells were incubated with a SARS-CoV-2 spike-specific rabbit monoclonal antibody (cat. no. 40592-R001, Sino Biological, Eschborn, Germany), followed by Alexa Fluor 555-conjugated anti-rabbit IgG (cat. no. A32732, Thermo Fisher Scientific, Waltham, MA, USA). Data were acquired on a BD LSR Fortessa flow cytometer (BD Biosciences) and analyzed using FlowJo^®^ software v10 (BD Biosciences).

For immunofluorescence microscopy, 1.8 × 10^5^ CD14^+^ monocytes were seeded into 8-well chamber slides (ibidi GmbH, Gräfelfing, Germany) and infected with ORFV-S or Jcovden at an MOI of 10, or left uninfected. After 16 h, cells were fixed with 4% methanol-free formaldehyde (Thermo Fisher Scientific) for 15 min at room temperature, permeabilized with 0.2% Triton X-100 in PBS for 5 min, and blocked with 5% BSA in PBS. Cells were then incubated for 2 h at room temperature with the spike-specific primary antibody (cat. no. 40592-R001, Sino Biological), followed by Alexa Fluor 555-conjugated anti-rabbit IgG (cat. no. A32732, Thermo Fisher Scientific). Nuclei were counterstained with NucBlue Live Cell Stain (Invitrogen, Thermo Fisher Scientific), and slides were mounted with ibidi Mounting Medium. Images were acquired using a Zeiss LSM800 confocal microscope with a 63× oil immersion objective and processed using ZEN Blue 3.0 software (Carl Zeiss Microscopy GmbH, Oberkochen, Germany).

### 2.3 Immunization

The ORFV-S was prepared as previously described (41). The Jcovden was purchased and used according to the manufacturer’s instructions. Female CD-1 mice (Charles River Laboratories, Sulzfeld, Germany), aged 7–9 weeks, were randomly assigned to experimental groups (n = 5–10 per group). We chose an outbred strain to better approximate genetic diversity and reduce strain-specific biases in humoral and T-cell readouts, which is common practice in vaccine immunogenicity screens (46). Mice were immunized intramuscularly (i.m.) on days 0 and 21 with either 1/10 of the human dose of Jcovden or 1 × 10^6^ plaque-forming units (PFU) of ORFV-S. The selected dose of ORFV-S corresponds to approximately 1/100 of the human dose (1 × 10^8^ PFU). The intramuscular route was chosen to reflect clinical use of both platforms and to enable a head-to-head immunogenicity comparison under clinically relevant conditions. Peripheral blood was collected from the tail vein under isoflurane anesthesia (3–4% in O_2_) on days 14 and 28. On day 28, a subset of animals was euthanized under isoflurane anesthesia by cardiac puncture, and spleens were harvested for downstream analyses. Splenocytes were isolated and cryopreserved according to standard protocols. At the final experimental time point (day 35), all remaining animals were anesthetized with isoflurane and euthanized by cardiac puncture; blood samples were subsequently collected.

### 2.4 Detection of specific serum IgG by ELISA

Spike-binding antibodies were quantified in mouse serum samples using an indirect enzyme-linked immunosorbent assay (ELISA). Nunc Maxisorp 96-well plates (Fisher Scientific, Schwerte, Germany) were coated overnight at 4°C with 5 μg/ml of recombinant SARS-CoV-2 full-length Spike protein (Cat. No. 40589-V08B1, Sino Biological) in PBS (Thermo Fisher Scientific). Plates were then blocked for 2 hours at room temperature with 3% bovine serum albumin (BSA; Carl Roth, Karlsruhe, Germany) in PBS to prevent non-specific binding. Afterwards, serial dilutions of serum samples were added to the wells and incubated for 1 hour at room temperature. Antigen-bound antibodies were detected using horseradish peroxidase (HRP)-conjugated goat anti-mouse antibodies specific for total IgG (1:5000, Abcam, Cambridge, UK, ab6728), IgG1 (1:1000, Abcam, ab97240), or IgG2a (1:1000, Abcam, ab97245). Following 1-hour incubation, plates were developed using TMB substrate (Cat. No. 421101, BioLegend) and the reaction was stopped after sufficient color development by adding stop solution (Cat. No. 423001, BioLegend). All samples and controls were run in duplicate. Absorbance was measured at 450 nm, and background values (blank wells) were subtracted from sample readings. Endpoint titers were determined by plotting the log_10_-transformed optical density (OD) values against the log_10_ of the serum dilution. A linear regression model was applied, and the endpoint titer was defined as the dilution at which the regression line of the sample intersected with the OD cut-off value of 0.1.

### 2.5 RBDCoV-ACE2 measurements

RBDCoV-ACE2, a previously published multiplex ACE2 inhibition assay (47), analyses neutralizing antibody activity using ACE2 binding inhibition as a surrogate. Neutralizing antibodies were analyzed against the SARS-CoV-2 WT, Beta, Delta, Omicron BA2 and XBB.1.5 variants. Samples were measured at dilution factor of 1:400 and 1:2400. In brief, RBD variant proteins (48–51) were coupled to spectrally distinct populations of MagPlex beads (cat. no. MC100XX, Luminex, Austin, TX, USA) and then combined into a bead mix. Serum samples were diluted with assay buffer and then ACE2 buffer (300 ng/mL biotinylated ACE2, cat. no. 10108-H08H-B, Sino Biological), before being combined 1:1 with the bead mix in 96 well plates (cat. no. 3642, Corning, Corning, NY, USA). After incubation for 2 hours at 21°C, 750rpm in a thermomixer, the beads were washed 3x in wash buffer using an automated microplate washer (Biotek 405TS, Winooski, VT, USA). Bound ACE2 was detected using 2ug/mL Strep-PE (cat. no. SAPE-001, Moss, Pasadena, MD, USA) by incubating the bead-sample mix for a further 45 mins. Following a further washing step, the beads were resuspended in 100uL of wash buffer, shaken for 3 mins at 1000rpm and then measured on a FLEXMAP3D using the following settings: Timeout 80 sec, Gate 7500-15000, Reporter Gain: Standard PMT, 50 events. As controls, 150ng/mL ACE2, blanks and 2 QC samples (all in duplicate) were included. ACE2 binding inhibition (%) was calculated as a percentage, with 100% indicating maximum ACE2 binding inhibition and 0% no ACE2 binding inhibition.

### 2.6 Intracellular cytokine staining

Cryopreserved splenocytes were thawed, rested for 4 hours at 37°C in complete RPMI medium, and seeded into 96-well round-bottom plates (Greiner Bio-One, Frickenhausen, Germany) at a density of 2 × 10^6^ cells per well. Cells were re-stimulated with 0.5 μg/mL of SARS-CoV-2 full-length spike (PM-WCPV-S-1) or nucleocapsid (PM-WCPV-NCAP-1) peptide pools (both JPT Peptide Technologies, Berlin, Germany) in the presence of 1 μg/mL anti-mouse CD28 (cat. no. 102116) and CD49d (cat. no. 103710) co-stimulatory antibodies (both BioLegend). After 1 hour of stimulation, Brefeldin A (10 μg/mL; Sigma-Aldrich, St. Louis, MO, USA), Monensin (cat. no. 420701, BioLegend), and anti-mouse CD107a antibody (cat. no. 121620, BioLegend) were added, and cells were incubated for an additional 14 hours at 37°C. Following stimulation, cells were blocked with TruStain FcX™ (anti-mouse CD16/32, cat. no. 101320, BioLegend) for 10 minutes at room temperature and subsequently stained for 30 minutes at room temperature with a surface antibody cocktail containing anti-CD3ϵ (cat. no. 100312), CD4 (cat. no. 100531), CD8α (cat. no. 100730), CD62L (cat. no. 104430), and CD44 (cat. no. 103022), together with Zombie Aqua™ Fixable Viability Dye (cat. no. 423102, all BioLegend). Cells were then fixed and permeabilized using Fixation & Permeabilization Solution (BD Biosciences) for 30 minutes and stained intracellularly for cytokines with anti-mouse TNF-α (cat. no. 506344), IFN-γ (cat. no. 505835), IL-2 (cat. no. 503808), IL-4 (cat. no. 504104), and IL-17A (cat. no. 506941) antibodies (all BioLegend) for 30 minutes at 4°C. Samples were acquired on an Attune NxT flow cytometer (Thermo Fisher Scientific) and analyzed using FlowJo^®^ v10 software (BD Biosciences). Background responses from unstimulated control wells were subtracted from peptide-stimulated conditions.

### 2.7 Germinal centre B cells and T follicular helper Cells

Cryopreserved splenocytes were thawed and rested for 4 hours at 37°C in complete RPMI medium. To block Fc receptors, cells were incubated with TruStain FcX™ (anti-mouse CD16/32, cat. no. 101320, BioLegend) for 10 minutes at room temperature. Surface staining was then performed for 30 minutes at 4°C using an antibody cocktail targeting the following markers: CD3ϵ (cat. no. 100320), CD4 (cat. no. 100531), CD8α (cat. no. 100730), CD62L (cat. no. 104406), CD44 (cat. no. 103032), CD19 (cat. no. 115530), CXCR5 (cat. no. 145529), CD95 (cat. no. 152604), and GL7 (cat. no. 144608), along with Zombie Aqua™ Fixable Viability Dye (cat. no. 423102, all BioLegend). Samples were acquired on an Attune NxT flow cytometer (Thermo Fisher Scientific) and analyzed using FlowJo^®^ v10 software (BD Biosciences). Germinal center CD4+ T follicular helper (GC Tfh) cells in spleen were identified as CD4+ CD44^high^CD62L^low^ CXCR5^high^ GL7+ (52). Germinal center B cells (GC B cells) in spleen were identified as CD19+ CD95+ GL7+ (52).

### 2.8 Statistical analysis

Statistical analyses were performed using GraphPad Prism version 9 (GraphPad Software, San Diego, CA, USA). Normality of data distributions was assessed using the Shapiro–Wilk test. Group comparisons were conducted using the Mann–Whitney U test or Kruskal–Wallis test, as appropriate. P-values < 0.05 were considered statistically significant. Statistical significance is indicated as follows: *p < 0.05; **p < 0.01. Data are presented as means ± standard error of the mean (SEM), geometric mean with geometric standard deviation (SD), or medians, as indicated in the figure legends.

## 3 Results

### 3.1 Spike antigen expression from ORFV-S and Jcovden vaccines

Both vaccines encode the full-length, prefusion-stabilized spike protein of the ancestral Wuhan SARS-CoV-2 strain (27, 41, 53, 54). ORFV-S incorporates the D614G substitution, the K986P/V987P double proline mutations, and a GSAS replacement of the polybasic furin cleavage site (RRAR, residues 682–685) (41), mirroring the stabilized spike design used in Jcovden (27).

We compared spike antigen expression mediated by the ORFV-based vaccine vector (ORFV-S) and the adenoviral vector Jcovden (Ad26.COV2.S) in HEK293-derived VPC 2.0 cells and in human PBMC-derived CD14^+^ monocytes across a range of MOIs.

**Figure 1:**
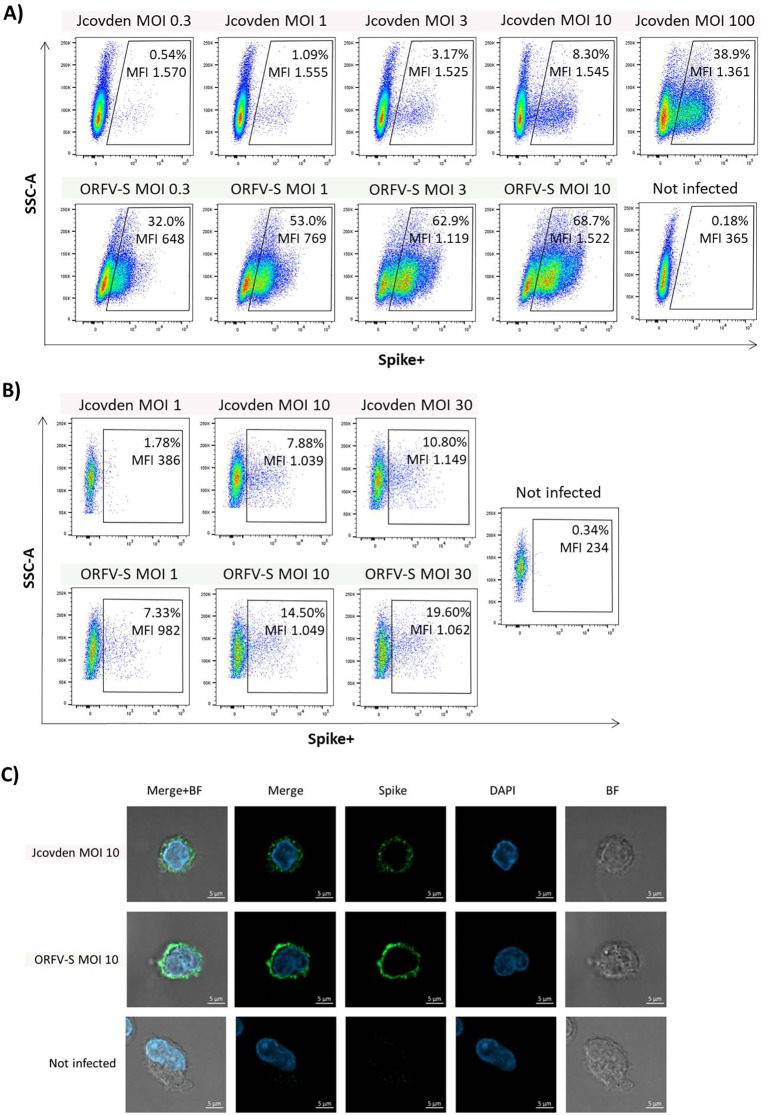
Spike antigen expression following ORFV-S and Jcovden infection. HEK293-derived VPC 2.0 cells and human CD14^+^ monocytes cells were infected with ORFV-S and Jcovden at indicated multiplicity of infection (MOI) for 16 h Not infected cells served as controls. Expression of spike protein was analyzed on the cell surface using specific anti-spike S1 antibody. **(A, B)** Flow cytometry analysis of **(A)** VPC 2.0 cells and **(B)** CD14+ monocytes of all viable cells. Mean fluorescence intensity (MFI) values are shown for the samples. **(C)** Fluorescence microscopy images of antigen expression in CD14+ monocytes.

Flow cytometry analysis revealed that ORFV-S induced robust spike expression already at a low MOI of 0.3, with ~32% of VPC 2.0 cells staining positive for surface spike protein. The frequency of spike-positive cells further increased with higher MOIs (**Figure 1A**). In contrast, a comparable percentage of spike-positive cells was observed with Jcovden only at a MOI of 100. This difference may reflect the substantially higher particle dose used in the human Jcovden vaccine (approximately 500-fold higher than ORFV-based formulations).

Comparable results were obtained in primary antigen-presenting cells. Infection of CD14^+^ monocytes with ORFV-S at MOIs 1 and 10 resulted in approximately two-fold higher frequencies of spike-positive cells compared with Jcovden at the same MOIs (**Figure 1B**).

These flow cytometry findings were corroborated by immunofluorescence microscopy, which demonstrated stronger spike-specific fluorescence on the surface of CD14^+^ monocytes infected with ORFV-S at MOI 10 compared with Jcovden at the same MOI (**Figure 1C**).

### 3.2 Spike-specific IgG responses

**Figure 2:**
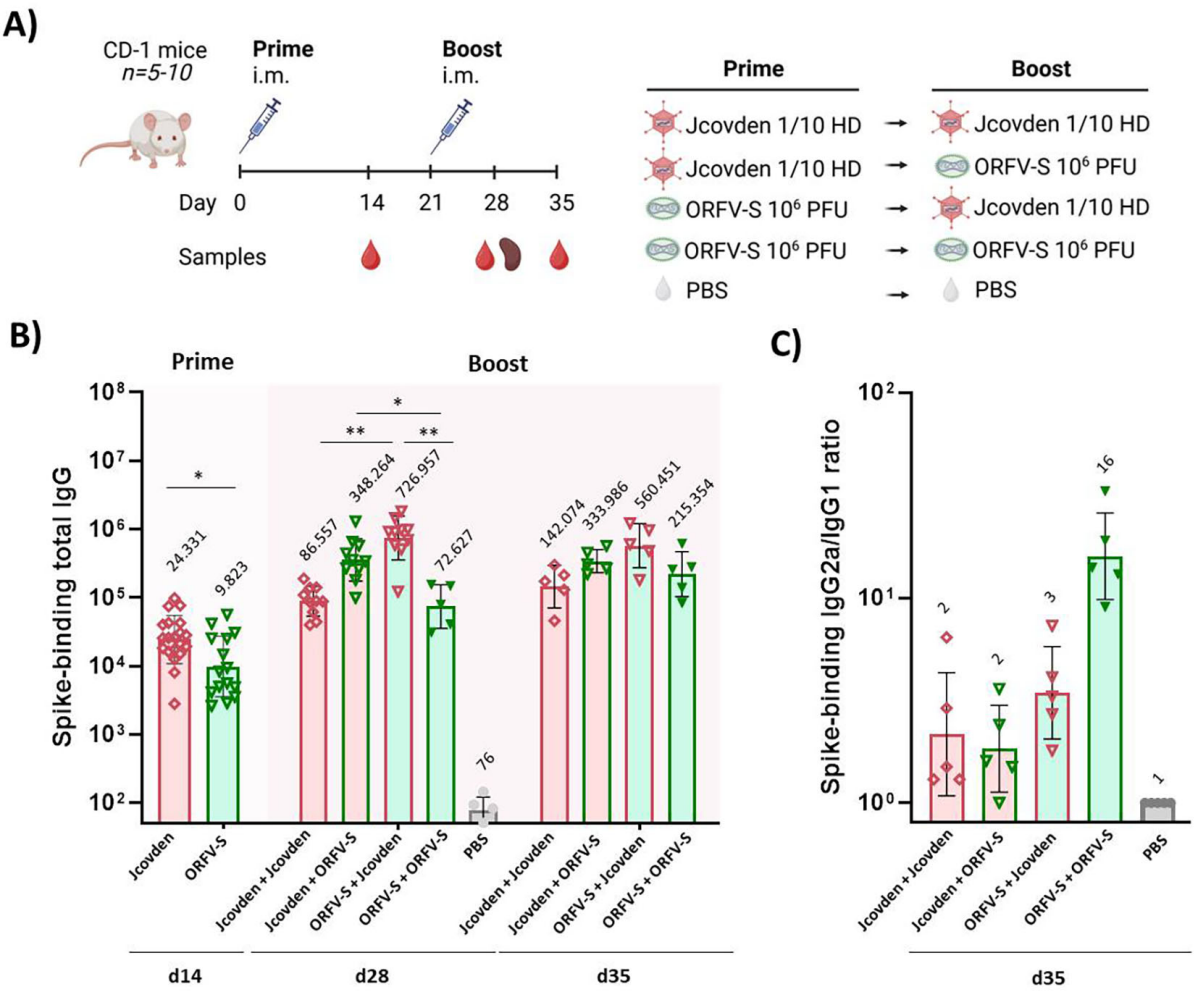
Humoral immune responses following homologous and heterologous immunization with ORFV-S and Jcovden in CD-1 mice. **(A)** Schematic overview of the experimental design, including group allocation, vaccination schedule, and sample collection time points. **(B)** Endpoint titers of spike-specific total IgG in mouse serum measured by ELISA at 2 weeks after the first immunization (day 14) and 1 and 2 weeks after the second immunization (days 28 and 35). Data for day 14 were obtained from all mice prior to group allocation into homologous or heterologous boost regimens and therefore represent pooled values for the same cohort. **(C)** Ratio of IgG2a to IgG1 isotypes among spike-specific antibodies on day 35, indicating Th1/Th2 polarization. Geometric mean titers (GMT) are noted above the columns. *p < 0.05; **p < 0.01.

Mice were immunized with either homologous or heterologous vaccine regimens, and spike-specific IgG titers were quantified by ELISA (**Figure 2A**). A single immunization with 10^6^ PFU of ORFV-S—equivalent to approximately 1/100 of the intended human dose—induced significantly lower spike-specific IgG titers two weeks post-vaccination compared to a 1/10 human dose of Jcovden (**Figure 2B**).

Heterologous prime-boost combinations outperformed homologous regimens across both platforms. Notably, animals primed with ORFV-S and boosted with Jcovden exhibited the highest levels of spike-specific IgG. The reverse sequence—Jcovden prime followed by ORFV-S boost—also improved responses relative to homologous Jcovden immunization, albeit to a lesser extent. These findings demonstrate the immunological advantage of heterologous prime-boost approaches (55–57) and highlight ORFV-S as a potent vector capable of effectively priming and boosting humoral responses in combination with licensed adenoviral vaccine.

### 3.3 IgG subclass profiles and Th1 bias

To evaluate the qualitative nature of the antibody responses, we analyzed IgG subclass distributions in serum samples collected two weeks after booster immunization. The IgG2a/IgG1 ratio serves as a commonly used indicator of Th1- versus Th2-skewed immunity (58–60).

All vaccination regimens induced a predominant IgG2a response over IgG1, suggesting that both ORFV-S and Jcovden preferentially elicit Th1-biased humoral immunity, independent of vaccination sequence (**Figure 2C**). Given that Th1-type responses are associated with protection and reduced immunopathology in coronavirus infections (61–63), this immunological signature is favorable for both vaccine platforms and their combination.

### 3.4 ACE2 binding inhibition of SARS-CoV-2 variants of concern

To assess the functional activity of vaccine-induced antibodies, we performed an RBDCoV-ACE2 inhibition assay to evaluate their capacity to block ACE2 binding to the receptor-binding domain (RBD) of SARS-CoV-2 variants.

**Figure 3:**
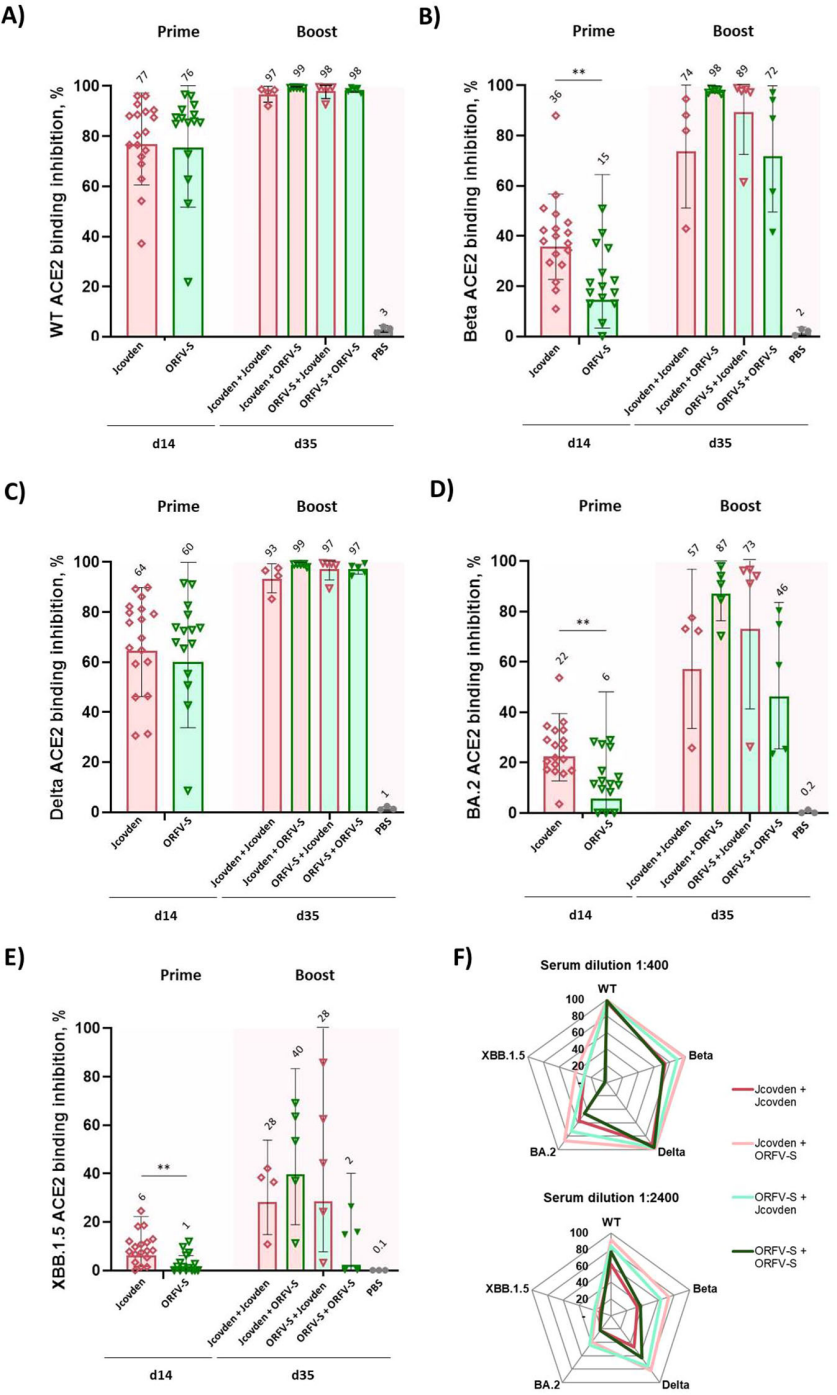
ACE2 binding inhibition against SARS-CoV-2 variants of concern (VoC) following homologous and heterologous immunization with ORFV-S and Jcovden in CD-1 mice, assessed by the RBDCoV-ACE2 assay. ACE2 binding inhibition (%) in serum was measured at two time points: day 14 (two weeks after the first immunization) and day 35 (two weeks after the second immunization). Data for day 14 were obtained from all mice prior to group allocation into homologous or heterologous boost regimens and therefore represent pooled values for the same cohort. Inhibition was assessed against the following RBD variants: **(A)** Wild-type (WT), **(B)** Beta, **(C)** Delta, **(D)** Omicron BA.2, and **(E)** Omicron XBB.1.5. Data are presented as geometric mean values ± geometric SD. GMT are indicated above the bars. **(F)** Radar chart displaying geometric mean ACE2 binding inhibition (%) in serum on day 35 across all five variants. In **(A–E)**, results are shown at a fixed serum dilution of 1:400; in **(F)**, at dilutions of 1:400 and 1:2400. Responses <0.1% were set to 0.1% for visualization purposes; values >20% are considered indicative of a positive response. **p < 0.01.

Two weeks after a single immunization, sera from animals receiving 10^6^ PFU of ORFV-S showed robust ACE2 binding inhibition against the ancestral (wild-type, WT) and Delta variants, comparable to that induced by 1/10 human dose of Jcovden (**Figures 3A, C**). Notably, this occurred despite significantly higher spike-binding IgG titers in the Jcovden group, suggesting that ORFV-S elicits functionally potent antibodies even at low doses, reflecting the strong immunogenicity of the ORFV platform.

All prime-boost regimens elicited high levels of ACE2 binding inhibition against pre-Omicron variants (WT, Beta, and Delta), with responses plateauing for WT and Delta (**Figures 3A–C**). For the immune-evasive Beta variant, heterologous regimens were clearly superior to homologous ones. The strongest inhibition was observed in animals primed with Jcovden and boosted with ORFV-S, indicating a synergistic effect. Boosting ORFV-S–primed animals with Jcovden also enhanced responses, though to a lesser extent, underscoring the superior boosting capacity of ORFV-S.

As expected, ACE2 binding inhibition against Omicron-related variants was reduced across all groups (**Figures 3D–F**). In this setting, a single immunization with ORFV-S was less effective than 1/10 human dose of Jcovden. Nevertheless, heterologous prime-boost combinations again outperformed homologous regimens, with the Jcovden–ORFV-S sequence yielding the highest inhibition levels (**Figure 3F**). These findings further highlight the breadth and potency of the antibody responses elicited by the heterologous prime-boost approach.

### 3.5 Germinal center B cells and Tfh cell responses

Germinal center (GC) B cells and T follicular helper (Tfh) cells are key components of the adaptive immune response, contributing to the development of high-affinity antibodies and long-lived humoral memory (64, 65). To evaluate the impact of different vaccination regimens on these cell populations, we evaluated the presence of GC B cells and their interaction with Tfh cells in spleen one week after booster immunization (**Supplementary Figure S1A**).

All vaccine regimens—regardless of platform or sequence—induced comparable frequencies of GC B cells and Tfh cells. These findings suggest that both ORFV-S and Jcovden support the cellular infrastructure required for affinity maturation and sustained antibody responses, independent of the vaccination strategy.

### 3.6 Spike-specific CD4^+^ and CD8^+^ T cell responses

To further characterize the cellular immune responses elicited by the different vaccine regimens, we analyzed spike-specific CD4^+^ and CD8^+^ T cells in splenocytes by intracellular cytokine staining (ICS) following *ex vivo* restimulation with spike peptide pools.

**Figure 4:**
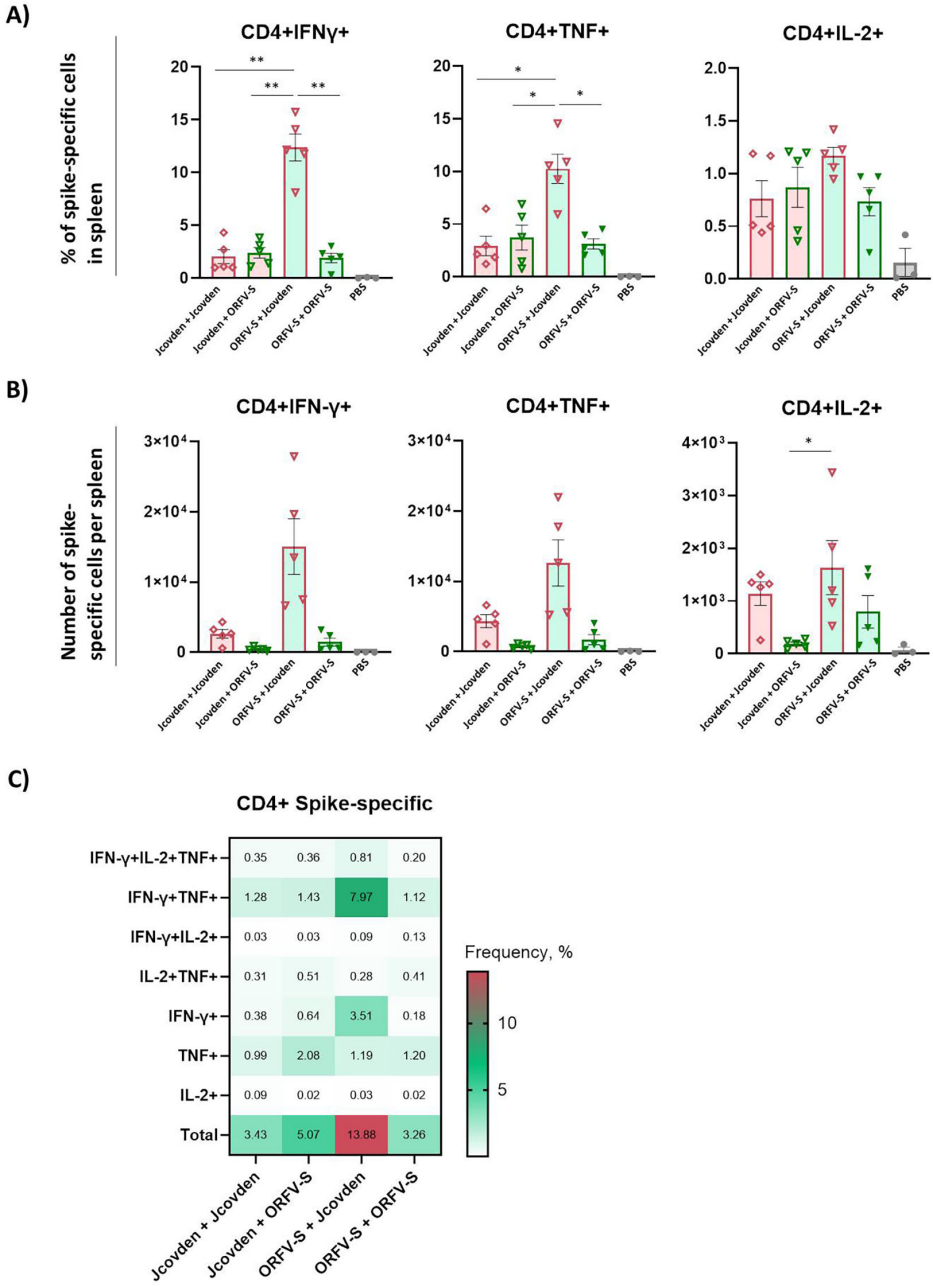
Antigen-specific CD4^+^ T cell immune responses induced by homologous and heterologous vaccination with ORFV-S and Jcovden in CD-1 mice. CD-1 mice were immunized on days 0 and 21 with 10^6^ PFU of ORFV-S, 1/10 of the human dose of Jcovden, or PBS as control. Splenocytes were harvested on day 28 and stimulated *ex vivo* with SARS-CoV-2 spike peptide pools, followed by intracellular cytokine staining (ICS). **(A)** Percentage and **(B)** absolute number of cytokine-producing CD4^+^ T cells per spleen. Heights of bars indicate mean ± SEM (standard error of the mean). **(C)** Heatmap showing mean frequencies of polyfunctional spike-specific CD4^+^ T cells per group. *p < 0.05; **p < 0.01.

Both homologous prime-boost regimens induced comparable frequencies and absolute numbers of spike-specific CD4^+^ T cells (**Figures 4A, B**). The heterologous combination of ORFV-S prime followed by Jcovden boost resulted in the highest CD4^+^ T cell responses, whereas the reverse sequence was less effective.

Consistent with the observed IgG2a/IgG1 ratios, CD4^+^ T cell cytokine profiles revealed a Th1-biased phenotype characterized by elevated IFN-γ, TNF, and IL-2 production, with low levels of IL-4 and IL-17A. Interestingly, IL-4 levels were slightly elevated in groups that received Jcovden, which may reflect vector-intrinsic differences in innate activation or antigen presentation compared with the ORFV platform (**Supplementary Figure S1B**).

Although the ORFV-S prime followed by Jcovden boost regimen elicited the highest spike-specific CD4^+^ T cell frequencies and IgG titers on day 28, no correlation was observed between these two parameters (**Supplementary Figure S1C**), suggesting that cellular and humoral responses may be regulated independently or follow distinct kinetics.

We next examined the quality of Th1-related CD4^+^ T cell responses by analyzing polyfunctionality, defined as the simultaneous production of multiple effector cytokines by individual cells. Among spike-specific CD4^+^ T cells, the ORFV-S prime/Jcovden boost group showed the highest frequencies of dual-positive IFN-γ^+^/TNF^+^ cells as well as IFN-γ^+^ single-positive cells (**Figure 4C**).

**Figure 5:**
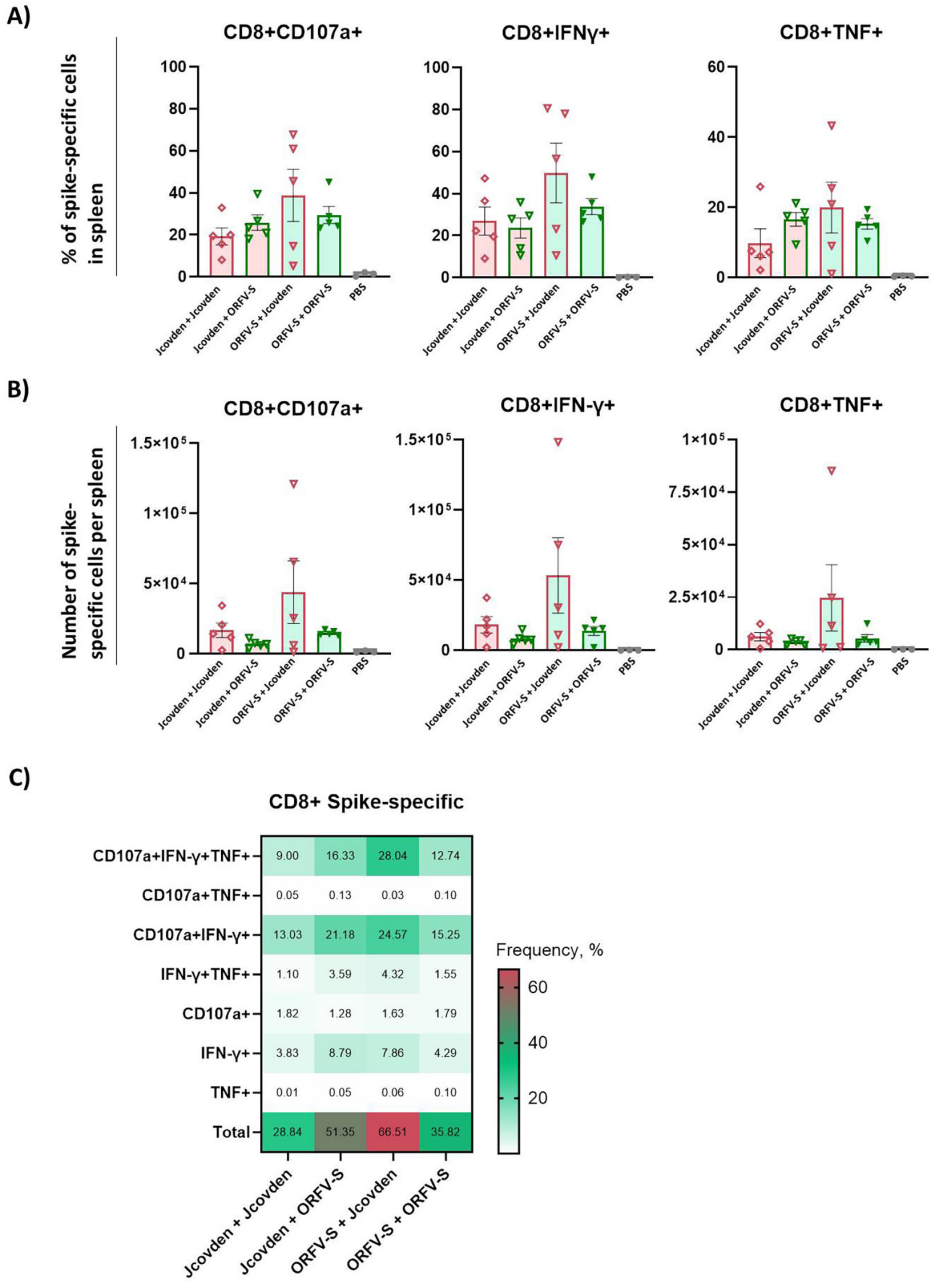
Antigen-specific CD8^+^ T cell immune responses induced by homologous and heterologous vaccination with ORFV-S and Jcovden in CD-1 mice. CD-1 mice were immunized on days 0 and 21 with 10^6^ PFU of ORFV-S, 1/10 of the human dose of Jcovden, or PBS as control. Splenocytes were harvested on day 28 and stimulated *ex vivo* with SARS-CoV-2 spike peptide pools, followed by intracellular cytokine staining (ICS). **(A)** Percentage and **(B)** absolute number of cytokine-producing CD8^+^ T cells per spleen. Heights of bars indicate mean ± SEM (standard error of the mean). **(C)** Heatmap showing mean frequencies of polyfunctional spike-specific CD8^+^ T cells per group.

Consistent with the findings for CD4^+^ T cell responses, a similar pattern was observed for spike-specific CD8^+^ T cells (**Figures 5A, B**). The heterologous regimen with ORFV-S prime followed by Jcovden boost induced the most robust CD8^+^ T cell responses, surpassing both homologous combinations. In contrast, the reverse sequence—Jcovden prime followed by ORFV-S boost—did not enhance CD8^+^ T cell responses and resulted in lower levels than those seen with either homologous regimen.

For CD8^+^ T cells, polyfunctionality analysis revealed a high proportion of triple-positive CD107a^+^/IFN-γ^+^/TNF^+^ cells, particularly in the heterologous ORFV-S + Jcovden group (**Figure 5C**). The homologous ORFV-S regimen also elicited high levels of these polyfunctional CD8^+^ T cells, highlighting the strong cytotoxic potential of the ORFV-based platform.

## 4 Discussion

This study investigated whether the distinct profiles of an ORFV-based spike vaccine (ORFV-S) and the licensed Ad26 vector vaccine Jcovden (Ad26.COV2.S) could be harnessed in heterologous prime–boost regimens to enhance SARS-CoV-2 immunity. Leveraging spike as a clinically relevant and immunologically well-characterized antigen, we systematically compared homologous and mixed-platform schedules in mice to determine how two mechanistically divergent vectors influence the magnitude, quality, and breadth of vaccine-induced immunity.

The 1/10 human dose of Jcovden was selected as the maximal feasible injection volume for mice, ensuring adequate vaccine delivery without injection-related adverse effects. In contrast, a 1/100 human dose of ORFV-S was intentionally used as a sub-immunogenic dose. Previous studies in mice (41) showed that higher ORFV-S doses rapidly reach plateau levels of spike-specific antibodies, thereby masking potential differences between homologous and heterologous regimens. Using a sub-dose therefore enabled a more sensitive assessment of heterologous prime–boost effects on the magnitude and quality of immune responses and were selected to produce measurable yet distinguishable responses rather than equivalent spike protein expression levels.

Head-to-head *in vitro* and *in vivo* analysis illuminated each vector’s strengths and limits. Jcovden is administered at a markedly higher particle dose compared to ORFV-S (approx. 500-fold higher) (29), and consistently, higher MOIs of Jcovden were required *in vitro* to achieve spike antigen expression levels comparable to ORFV-S in both VPC 2.0 cell line and primary CD14^+^ monocytes. The observed differences in spike protein expression at equivalent MOIs are likely platform-dependent, reflecting differences in cell susceptibility, infection efficiency, and regulatory elements influencing transgene expression, such as promoter strength, transcriptional or translational control, and spike surface presentation. We did not quantify *in vivo* spike expression, as such measurements would require extensive tissue-specific analysis of vector tropism and antigen-presentation kinetics. Given the different biology of ORFV and Ad26, expression levels are not expected to be directly comparable or predictive of the distinct immune outcomes observed.

The higher particle dose of Jcovden is consistent with its strong priming capacity *in vivo*, where it induced elevated spike-specific IgG levels early after vaccination, reflecting its ability to drive robust humoral responses after a single dose (29). However, a second Ad26 injection raised geometric mean titres by only ~6-fold, indicating recall is curtailed by anti-vector immunity (33, 66, 67). ORFV-S, by contrast, primed lower antibody levels but proved highly boost-responsive, with a ~22-fold increase after the second dose. Both homologous regimens generated comparable CD4^+^-T-cell frequencies (~3.3–3.4%) and robust CD8^+^ responses, with ORFV-S trending higher (35.8% vs 28.8%). Cytokine profiles were similarly polyfunctional, highlighting their capacity to support antiviral cellular immunity (68, 69). Together, these data suggest that Jcovden is a potent priming vector but sub-optimal booster, whereas ORFV retains high boostability.

The complementary features of the two platforms provided a rationale for heterologous pairing, and indeed the mixed regimens outperformed both homologous schedules. ORFV-S → Jcovden produced the highest spike-binding IgG titers and vigorous CD4^+^ and CD8^+^ responses, with a dominant Th1 cytokine signature that is considered crucial for antiviral protection (70). Conversely, Jcovden → ORFV-S elicited the strongest functional antibody responses, with superior ACE2-binding inhibition across multiple SARS-CoV-2 variants, including immune-evasive strains such as Beta, BA.2, and XBB.1.5. Notably, these functional responses were not directly proportional to total IgG titers, indicating that ORFV-S boosting promotes qualitative improvements in antibody affinity and neutralization breadth. These findings suggest that sequence-dependent effects allow heterologous schedules to emphasize either cellular or humoral arms of the adaptive response.

The enhanced immunity likely reflects engagement of complementary innate and adaptive pathways. While both Ad26 and ORFV ultimately converge on cGAS–STING-dependent DNA sensing (19, 33, 42, 43), yet they may differ in other immunostimulatory properties that likely underlie the sequence-dependent synergy observed here. Ad26 vectors activate Th1-biased responses through cGAS–STING and RIG-I/MAVS (19, 33), whereas ORFV provides evidence of a broader spectrum of innate activation, including cytosolic RNA sensors (RIG-I, MDA5, PKR) and DAMP/PAMP signatures inherent to its virions (42, 43). This may translate into stronger activation of antigen-presenting cells and more efficient cross-priming of T cells. Furthermore, platform-specific differences in antigen expression kinetics and tropism for dendritic cell subsets could amplify complementary pathways of B- and T-cell activation. Together, these mechanistic features may offer an explanation for the non-redundant and sequence-dependent synergy observed in this study.

Our results resonate with an established body of evidence supporting heterologous viral vector vaccination. An Ad5 prime–MVA boost strategy targeting the tuberculosis antigen Ag85A elicited significantly enhanced CD4^+^ and CD8^+^ T cell responses in mice compared to homologous Ad5 or MVA regimens (24). Similarly, in a malaria vaccine model, a heterologous Ad prime followed by an MVA boost induced not only higher frequencies of CD8^+^ T cells but also more durable and polyfunctional responses, surpassing those generated by both Ad–Ad and heterologous Ad–Ad combinations (25, 71). Additionally, for HIV and malaria antigens, heterologous regimens using Ad26–MVA or chimpanzee adenovirus–MVA combinations elicited stronger and longer-lasting cellular immunity compared to homologous vaccination protocols (25, 71, 72). Importantly, the exceptionally high CD8^+^ T-cell frequencies observed with ORFV–Ad26 combinations in our study suggest that this particular pairing may provide an especially powerful means of enhancing cytotoxic immunity beyond what has been achieved with previously tested adenovirus–poxvirus combinations.

Although SARS-CoV-2 served as the model antigen here, the broader implications extend well beyond COVID-19. Robust CD8^+^ T-cell responses, supported by Th1-biased CD4^+^ immunity, are increasingly recognized as central for vaccines against HIV, tuberculosis, malaria, CMV, and emerging zoonoses. ORFV-based vectors may hold unique advantages in this context, as they can be repeatedly administered without loss of potency, making them especially suitable for iterative vaccination strategies or outbreak scenarios requiring rapid redeployment. The ability of ORFV vectors to efficiently boost antibody quality while simultaneously promoting cytotoxic T-cell responses positions them as promising partners in heterologous prime–boost regimens not only for infectious diseases but also for cancer immunotherapy.

Future studies could extend these findings in several important directions. While the murine model provides valuable insights into comparative immunogenicity, human studies will be needed to fully capture the complexity of vaccine-induced immunity. Evaluating protective efficacy in challenge models, as well as assessing the long-term durability of responses, will be key to establishing translational relevance. It will also be important to quantify airway mucosal immunity and to explore targeted intranasal boosting strategies, given their potential to enhance protection at the primary site of viral entry. In addition, mechanistic work dissecting innate sensing and antigen presentation pathways could help explain the sequence-dependent effects observed. Ultimately, clinical evaluation will be essential to confirm the potential of heterologous ORFV–Ad26 regimens.

In conclusion, our data demonstrate that ORFV- and Ad26-based vectors engage the immune system through complementary mechanisms, and that their heterologous use substantially enhances both the magnitude and quality of vaccine-induced immunity. Rational pairing of viral vectors can therefore be leveraged to fine-tune immune responses, offering a versatile approach for the design of next-generation vaccines against diverse pathogens and potentially cancer.

## Data Availability

The original contributions presented in the study are included in the article/**Supplementary Material**. Further inquiries can be directed to the corresponding author.

## Supplementary material

Supplementary Figure 1 Germinal center and antigen-specific CD4^+^ T cell responses induced by ORFV-S and Jcovden in homo- and heterologous vaccination regimens. CD-1 mice were immunized on days 0 and 21 with 10^6^ PFU of ORFV-S, 1/10 of the human dose of Jcovden, or PBS. **(A)** Number of germinal center (GC) B cells and T follicular helper (Tfh) cells per spleen on day 28. **(B)** Number of spike-specific CD4^+^ T cells per spleen assessed on day 28 by intracellular cytokine staining (ICS) following *ex vivo* peptide restimulation. Heights of bars indicate mean ± SEM (standard error of the mean). **(C)** Correlation between the numbers of antigen-specific CD4^+^ T cells per spleen and the corresponding total IgG endpoint titer in serum on day 28.

## References

[1] Wang S Li W Wang Z Yang W Li E Xia X . Emerging and reemerging infectious diseases: global trends and new strategies for their prevention and control. Signal Transduct Target Ther. (2024) 9:223. doi: 10.1038/s41392-024-01917-x, PMID: 39256346 PMC11412324

[2] Jacobsen H Sitaras I Katzmarzyk M Cobos Jimenez V Naughton R Higdon MM . Systematic review and meta-analysis of the factors affecting waning of post-vaccination neutralizing antibody responses against sars-cov-2. NPJ Vaccines. (2023) 8:159. doi: 10.1038/s41541-023-00756-1, PMID: 37863890 PMC10589259

[3] Zou Y Lo WC Ming WK Yuan HY . Impact of vaccination on omicron's escape variants: insights from fine-scale modelling of waning immunity in hong kong. Infect Dis Model. (2025) 10:129–38. doi: 10.1016/j.idm.2024.09.006, PMID: 39380722 PMC11459622

[4] Carabelli AM Peacock TP Thorne LG Harvey WT Hughes J Consortium C-GU . Sars-cov-2 variant biology: immune escape, transmission and fitness. Nat Rev Microbiol. (2023) 21:162–77. doi: 10.1038/s41579-022-00841-7, PMID: 36653446 PMC9847462

[5] Tian J Shang B Zhang J Guo Y Li M Hu Y . T cell immune evasion by sars-cov-2 jn.1 escapees targeting two cytotoxic T cell epitope hotspots. Nat Immunol. (2025) 26:265–78. doi: 10.1038/s41590-024-02051-0, PMID: 39875585

[6] Markov PV Ghafari M Beer M Lythgoe K Simmonds P Stilianakis NI . The evolution of sars-cov-2. Nat Rev Microbiol. (2023) 21:361–79. doi: 10.1038/s41579-023-00878-2, PMID: 37020110

[7] Low ZY Wong KH Wen Yip AJ Choo WS . The convergent evolution of influenza a virus: implications, therapeutic strategies and what we need to know. Curr Res Microb Sci. (2023) 5:100202. doi: 10.1016/j.crmicr.2023.100202, PMID: 37700857 PMC10493511

[8] Nielsen BF Berrig C Grenfell BT Andreasen V . One hundred years of influenza a evolution. Theor Popul Biol. (2024) 159:25–34. doi: 10.1016/j.tpb.2024.07.005, PMID: 39094981

[9] Andrews SM Rowland-Jones S . Recent advances in understanding hiv evolution. F1000Res. (2017) 6:597. doi: 10.12688/f1000research.10876.1, PMID: 28529718 PMC5414815

[10] Druelle V Neher RA . Reversions to consensus are positively selected in hiv-1 and bias substitution rate estimates. Virus Evol. (2023) 9:veac118. doi: 10.1093/ve/veac118, PMID: 36632482 PMC9829961

[11] Hsiung KC Chiang HJ Reinig S Shih SR . Vaccine strategies against rna viruses: current advances and future directions. Vaccines (Basel). (2024) 12(12):1345. doi: 10.3390/vaccines12121345, PMID: 39772007 PMC11679499

[12] Sette A Saphire EO . Inducing broad-based immunity against viruses with pandemic potential. Immunity. (2022) 55:738–48. doi: 10.1016/j.immuni.2022.04.010, PMID: 35545026 PMC10286218

[13] Clemente-Suárez VJ Redondo-Flórez L Bustamante-Sánchez A Martín-Rodríguez A Yáñez-Sepúlveda R Tornero-Aguilera JF . Biometric strategies to improve vaccine immunogenicity and effectiveness. Biomimetics. (2025) 10:439. doi: 10.3390/biomimetics10070439, PMID: 40710252 PMC12292147

[14] Livieratos A Gogos C Thomas I Akinosoglou K . Vaccination strategies: mixing paths versus matching tracks. Vaccines (Basel). (2025) 13(3):308. doi: 10.3390/vaccines13030308, PMID: 40266207 PMC11946528

[15] Shirley JL De Jong YP Terhorst C Herzog RW . Immune responses to viral gene therapy vectors. Mol Ther. (2020) 28:709–22. doi: 10.1016/j.ymthe.2020.01.001, PMID: 31968213 PMC7054714

[16] Reguzova A Haug V Metz C Müller M Fandrich M Dulovic A . Heterologous prime-boost vaccination with vla2001 and an orfv-based vector enhances spike- and nucleocapsid-specific immunity in mice. Front Immunol. (2025) 16. doi: 10.3389/fimmu.2025.1675859, PMID: 41050708 PMC12488559

[17] Mai F Reisinger EC Muller-Hilke B . The type of the first prime/boost vaccine against sars-cov-2 exerts long-term effects on the humoral immune response. Clin Immunol. (2025) 278:110523. doi: 10.1016/j.clim.2025.110523, PMID: 40381868

[18] Jeon JH Kim S Kim SY Shin KS Park B Chang S . Heterologous prime-boost vaccination drives stromal activation and adaptive immunity against sars-cov-2 variants. Front Immunol. (2025) 16:1597417. doi: 10.3389/fimmu.2025.1597417, PMID: 40503231 PMC12151836

[19] Provine NM Klenerman P . Adenovirus vector and mrna vaccines: mechanisms regulating their immunogenicity. Eur J Immunol. (2023) 53:e2250022. doi: 10.1002/eji.202250022, PMID: 36330560 PMC9877955

[20] Isogawa M Onodera T Ainai A Kotaki R Kanno T Saito S . Prolonged effects of adenoviral vector priming on T-cell cytokine production in heterologous adenoviral vector/mrna covid-19 vaccination regimens. Sci Rep. (2025) 15:18684. doi: 10.1038/s41598-025-00054-x, PMID: 40436912 PMC12120080

[21] Piano Mortari E Ferrucci F Zografaki I Carsetti R Pacelli L . T and B cell responses in different immunization scenarios for covid-19: A narrative review. Front Immunol. (2025) 16:1535014. doi: 10.3389/fimmu.2025.1535014, PMID: 40170841 PMC11959168

[22] Logunov DY Dolzhikova IV Shcheblyakov DV Tukhvatulin AI Zubkova OV Dzharullaeva AS . Safety and efficacy of an rad26 and rad5 vector-based heterologous prime-boost covid-19 vaccine: an interim analysis of a randomised controlled phase 3 trial in Russia. Lancet. (2021) 397:671–81. doi: 10.1016/S0140-6736(21)00234-8, PMID: 33545094 PMC7852454

[23] Happe M Hofstetter AR Wang J Yamshchikov GV Holman LA Novik L . Heterologous cad3-ebola and mva-ebolaz vaccines are safe and immunogenic in us and Uganda phase 1/1b trials. NPJ Vaccines. (2024) 9:67. doi: 10.1038/s41541-024-00833-z, PMID: 38553525 PMC10980745

[24] Betts G Poyntz H Stylianou E Reyes-Sandoval A Cottingham M Hill A . Optimising immunogenicity with viral vectors: mixing mva and hadv-5 expressing the mycobacterial antigen ag85a in a single injection. PloS One. (2012) 7:e50447. doi: 10.1371/journal.pone.0050447, PMID: 23284637 PMC3528774

[25] Venkatraman N Anagnostou N Bliss C Bowyer G Wright D Lovgren-Bengtsson K . Safety and immunogenicity of heterologous prime-boost immunization with viral-vectored malaria vaccines adjuvanted with matrix-M. Vaccine. (2017) 35:6208–17. doi: 10.1016/j.vaccine.2017.09.028, PMID: 28941620

[26] Ratto-Kim S Currier JR Cox JH Excler J-L Valencia-Micolta A Thelian D . Heterologous prime-boost regimens using rad35 and rmva vectors elicit stronger cellular immune responses to hiv proteins than homologous regimens. PloS One. (2012) 7:e45840. doi: 10.1371/journal.pone.0045840, PMID: 23049876 PMC3458867

[27] Bos R Rutten L Van Der Lubbe JEM Bakkers MJG Hardenberg G Wegmann F . Ad26 vector-based covid-19 vaccine encoding a prefusion-stabilized sars-cov-2 spike immunogen induces potent humoral and cellular immune responses. NPJ Vaccines. (2020) 5:91. doi: 10.1038/s41541-020-00243-x, PMID: 33083026 PMC7522255

[28] Sadoff J Le Gars M Shukarev G Heerwegh D Truyers C De Groot AM . Interim results of a phase 1-2a trial of ad26.Cov2.S covid-19 vaccine. N Engl J Med. (2021) 384:1824–35. doi: 10.1056/NEJMoa2034201, PMID: 33440088 PMC7821985

[29] Sadoff J Gray G Vandebosch A Cardenas V Shukarev G Grinsztejn B . Safety and efficacy of single-dose ad26.Cov2.S vaccine against covid-19. N Engl J Med. (2021) 384:2187–201. doi: 10.1056/NEJMoa2101544, PMID: 33882225 PMC8220996

[30] Pollard AJ Launay O Lelievre JD Lacabaratz C Grande S Goldstein N . Safety and immunogenicity of a two-dose heterologous ad26.Zebov and mva-bn-filo ebola vaccine regimen in adults in europe (Ebovac2): A randomised, observer-blind, participant-blind, placebo-controlled, phase 2 trial. Lancet Infect Dis. (2021) 21:493–506. doi: 10.1016/S1473-3099(20)30476-X, PMID: 33217361

[31] Stieh DJ Barouch DH Comeaux C Sarnecki M Stephenson KE Walsh SR . Safety and immunogenicity of ad26-vectored hiv vaccine with mosaic immunogens and a novel mosaic envelope protein in hiv-uninfected adults: A phase 1/2a study. J Infect Dis. (2023) 227:939–50. doi: 10.1093/infdis/jiac445, PMID: 36348617 PMC10202119

[32] Salisch NC Stephenson KE Williams K Cox F Van Der Fits L Heerwegh D . A double-blind, randomized, placebo-controlled phase 1 study of ad26.Zikv.001, an ad26-vectored anti-zika virus vaccine. Ann Intern Med. (2021) 174:585–94. doi: 10.7326/M20-5306, PMID: 33587687

[33] Wang WC Sayedahmed EE Mittal SK . Significance of preexisting vector immunity and activation of innate responses for adenoviral vector-based therapy. Viruses. (2022) 14(12):2727. doi: 10.3390/v14122727, PMID: 36560730 PMC9787786

[34] Mendonca SA Lorincz R Boucher P Curiel DT . Adenoviral vector vaccine platforms in the sars-cov-2 pandemic. NPJ Vaccines. (2021) 6:97. doi: 10.1038/s41541-021-00356-x, PMID: 34354082 PMC8342436

[35] Zhang H Wang H An Y Chen Z . Construction and application of adenoviral vectors. Mol Ther Nucleic Acids. (2023) 34:102027. doi: 10.1016/j.omtn.2023.09.004, PMID: 37808925 PMC10556817

[36] Fleming SB Wise LM Mercer AA . Molecular genetic analysis of orf virus: A poxvirus that has adapted to skin. Viruses. (2015) 7:1505–39. doi: 10.3390/v7031505, PMID: 25807056 PMC4379583

[37] Buttner M Rziha HJ . Parapoxviruses: from the lesion to the viral genome. J Vet Med B Infect Dis Vet Public Health. (2002) 49:7–16. doi: 10.1046/j.1439-0450.2002.00539.x, PMID: 11911596

[38] Metz C Haug V Muller M Amann R . Pharmacokinetic and environmental risk assessment of prime-2-cov, a non-replicating orf virus-based vaccine against sars-cov-2. Vaccines (Basel). (2024) 12(5):492. doi: 10.3390/vaccines12050492, PMID: 38793743 PMC11126055

[39] Rziha HJ Buttner M Muller M Salomon F Reguzova A Laible D . Genomic characterization of orf virus strain D1701-V (Parapoxvirus) and development of novel sites for multiple transgene expression. Viruses. (2019) 11(2):127. doi: 10.3390/v11020127, PMID: 30704093 PMC6409557

[40] Haig DM Mercer AA . Ovine diseases. Orf Vet Res. (1998) 29:311–26. 9689744

[41] Reguzova A Muller M Pagallies F Burri D Salomon F Rziha HJ . A multiantigenic orf virus-based vaccine efficiently protects hamsters and nonhuman primates against sars-cov-2. NPJ Vaccines. (2024) 9:191. doi: 10.1038/s41541-024-00981-2, PMID: 39414789 PMC11484955

[42] AlDaif BA Fleming SB . Innate immune sensing of parapoxvirus orf virus and viral immune evasion. Viruses. (2025) 17(4):587. doi: 10.3390/v17040587, PMID: 40285029 PMC12031380

[43] Muller M Reguzova A Loffler MW Amann R . Orf virus-based vectors preferentially target professional antigen-presenting cells, activate the sting pathway and induce strong antigen-specific T cell responses. Front Immunol. (2022) 13:873351. doi: 10.3389/fimmu.2022.873351, PMID: 35615366 PMC9124846

[44] Esen M Fischer-Herr J Gabor JJ Gaile JM Fleischmann WA Smeenk GW . First-in-human phase I trial to assess the safety and immunogenicity of an orf virus-based covid-19 vaccine booster. Vaccines (Basel). (2024) 12(11):1288. doi: 10.3390/vaccines12111288, PMID: 39591190 PMC11599021

[45] Klinkardt U Schunk M Ervin J Schindler C Sugimoto D Rankin B . A novel orf virus vector-based covid-19 booster vaccine shows cross-neutralizing activity in the absence of anti-vector neutralizing immunity. Hum Vaccin Immunother. (2024) 20:2410574. doi: 10.1080/21645515.2024.2410574, PMID: 39397784 PMC11485980

[46] Thiriot J Liang Y Fisher J Walker DH Soong L . Host transcriptomic profiling of cd-1 outbred mice with severe clinical outcomes following infection with orientia tsutsugamushi. PloS Negl Trop Dis. (2022) 16:e0010459. doi: 10.1371/journal.pntd.0010459, PMID: 36417363 PMC9683618

[47] Junker D Dulovic A Becker M Wagner TR Kaiser PD Traenkle B . Covid-19 patient serum less potently inhibits ace2-rbd binding for various sars-cov-2 rbd mutants. Sci Rep. (2022) 12:7168. doi: 10.1038/s41598-022-10987-2, PMID: 35505068 PMC9062870

[48] Becker M Dulovic A Junker D Ruetalo N Kaiser PD Pinilla YT . Immune response to sars-cov-2 variants of concern in vaccinated individuals. Nat Commun. (2021) 12:3109. doi: 10.1038/s41467-021-23473-6, PMID: 34035301 PMC8149389

[49] Junker D Becker M Wagner TR Kaiser PD Maier S Grimm TM . Antibody binding and angiotensin-converting enzyme 2 binding inhibition is significantly reduced for both the ba.1 and Ba.2 Omicron Variants. Clin Infect Dis. (2023) 76:e240–e9. doi: 10.1093/cid/ciac498, PMID: 35717657 PMC9384292

[50] Woelfel S Dutschler J Konig M Dulovic A Graf N Junker D . Star sign study: evaluation of covid-19 vaccine efficacy against the sars-cov-2 variants bq.1.1 and xbb.1.5 in patients with inflammatory bowel disease. Aliment Pharmacol Ther. (2023) 58:678–91. doi: 10.1111/apt.17661, PMID: 37571863

[51] Woelfel S Dutschler J Junker D Konig M Leinenkugel G Graf N . Systemic and mucosal immunogenicity of monovalent xbb.1.5-adapted covid-19 mrna vaccines in patients with inflammatory bowel disease. Vaccines (Basel). (2024) 12(7):774. doi: 10.3390/vaccines12070774, PMID: 39066413 PMC11281571

[52] Tahiliani V Hutchinson TE Abboud G Croft M Salek-Ardakani S . Ox40 cooperates with icos to amplify follicular th cell development and germinal center reactions during infection. J Immunol. (2017) 198:218–28. doi: 10.4049/jimmunol.1601356, PMID: 27895177 PMC5173420

[53] Wu F Zhao S Yu B Chen YM Wang W Song ZG . A new coronavirus associated with human respiratory disease in China. Nature. (2020) 579:265–9. doi: 10.1038/s41586-020-2008-3, PMID: 32015508 PMC7094943

[54] Wrapp D Wang N Corbett KS Goldsmith JA Hsieh CL Abiona O . Cryo-em structure of the 2019-ncov spike in the prefusion conformation. Science. (2020) 367:1260–3. doi: 10.1126/science.abb2507, PMID: 32075877 PMC7164637

[55] Barros-Martins J Hammerschmidt SI Cossmann A Odak I Stankov MV Morillas Ramos G . Immune Responses against Sars-Cov-2 Variants after Heterologous and Homologous Chadox1 Ncov-19/Bnt162b2 Vaccination. Nat Med. (2021) 27:1525–9. doi: 10.1038/s41591-021-01449-9, PMID: 34262158 PMC8440184

[56] Hillus D Schwarz T Tober-Lau P Vanshylla K Hastor H Thibeault C . Safety, reactogenicity, and immunogenicity of homologous and heterologous prime-boost immunisation with Chadox1 ncov-19 and bnt162b2: A prospective cohort study. Lancet Respir Med. (2021) 9:1255–65. doi: 10.1016/S2213-2600(21)00357-X, PMID: 34391547 PMC8360702

[57] Spencer AJ McKay PF Belij-Rammerstorfer S Ulaszewska M Bissett CD Hu K . Heterologous vaccination regimens with self-amplifying rna and adenoviral covid vaccines induce robust immune responses in mice. Nat Commun. (2021) 12:2893. doi: 10.1038/s41467-021-23173-1, PMID: 34001897 PMC8129084

[58] Chung NH Chen YC Yang SJ Lin YC Dou HY Hui-Ching Wang L . Induction of Th1 and Th2 in the Protection against Sars-Cov-2 through Mucosal Delivery of an Adenovirus Vaccine Expressing an Engineered Spike Protein. Vaccine. (2022) 40:574–86. doi: 10.1016/j.vaccine.2021.12.024, PMID: 34952759 PMC8677488

[59] Prompetchara E Ketloy C Alameh MG Tharakhet K Kaewpang P Yostrerat N . Immunogenicity and protective efficacy of sars-cov-2 mrna vaccine encoding secreted non-stabilized spike in female mice. Nat Commun. (2023) 14:2309. doi: 10.1038/s41467-023-37795-0, PMID: 37085495 PMC10120480

[60] Mizuguchi Y Tsuzuki N Ebana MD Suzuki Y Kakuda T . Igg subtype response against virulence-associated protein a in foals naturally infected with rhodococcus equi. Vet Sci. (2024) 11(9):422. doi: 10.3390/vetsci11090422, PMID: 39330801 PMC11435873

[61] Graham BS . Rapid covid-19 vaccine development. Science. (2020) 368:945–6. doi: 10.1126/science.abb8923, PMID: 32385100

[62] Gil-Etayo FJ Garcinuno S Utrero-Rico A Cabrera-Marante O Arroyo-Sanchez D Mancebo E . An early th1 response is a key factor for a favorable covid-19 evolution. Biomedicines. (2022) 10(2):296. doi: 10.3390/biomedicines10020296, PMID: 35203509 PMC8869678

[63] Aleebrahim-Dehkordi E Molavi B Mokhtari M Deravi N Fathi M Fazel T . T helper type (Th1/th2) responses to sars-cov-2 and influenza a (H1n1) virus: from cytokines produced to immune responses. Transpl Immunol. (2022) 70:101495. doi: 10.1016/j.trim.2021.101495, PMID: 34774738 PMC8579696

[64] Victora GD Nussenzweig MC . Germinal centers. Annu Rev Immunol. (2022) 40:413–42. doi: 10.1146/annurev-immunol-120419-022408, PMID: 35113731

[65] Quast I . Tarlinton D. B cell memory: understanding covid-19. Immunity. (2021) 54:205–10. doi: 10.1016/j.immuni.2021.01.014, PMID: 33513337 PMC7826135

[66] Alter G Yu J Liu J Chandrashekar A Borducchi EN Tostanoski LH . Immunogenicity of ad26.Cov2.S vaccine against sars-cov-2 variants in humans. Nature. (2021) 596:268–72. doi: 10.1038/s41586-021-03681-2, PMID: 34107529 PMC8357629

[67] Mercado NB Zahn R Wegmann F Loos C Chandrashekar A Yu J . Single-shot ad26 vaccine protects against sars-cov-2 in rhesus macaques. Nature. (2020) 586:583–8. doi: 10.1038/s41586-020-2607-z, PMID: 32731257 PMC7581548

[68] Moss P . The T cell immune response against sars-cov-2. Nat Immunol. (2022) 23:186–93. doi: 10.1038/s41590-021-01122-w, PMID: 35105982

[69] Makedonas G Betts MR . Polyfunctional analysis of human T cell responses: importance in vaccine immunogenicity and natural infection. Springer Semin Immunopathol. (2006) 28:209–19. doi: 10.1007/s00281-006-0025-4, PMID: 16932955

[70] Sun L Su Y Jiao A Wang X . Zhang B. T cells in health and disease. Signal Transduct Target Ther. (2023) 8:235. doi: 10.1038/s41392-023-01471-y, PMID: 37332039 PMC10277291

[71] Reyes-Sandoval A Rollier CS Milicic A Bauza K Cottingham MG Tang CK . Mixed vector immunization with recombinant adenovirus and mva can improve vaccine efficacy while decreasing antivector immunity. Mol Ther. (2012) 20:1633–47. doi: 10.1038/mt.2012.25, PMID: 22354374 PMC3412496

[72] Ondondo BO . The influence of delivery vectors on hiv vaccine efficacy. Front Microbiol. (2014) 5:439. doi: 10.3389/fmicb.2014.00439, PMID: 25202303 PMC4141443

