# Supplementary material for: Heterologous ORFV–Ad26 vaccination broadens antibody breadth and amplifies cellular immunity against SARS-CoV-2 spike
Source: Front Immunol. 2025 Nov 18;16:1715442. doi: 10.3389/fimmu.2025.1715442 (PMC12669153; doi:10.3389/fimmu.2025.1715442)

**Supplementary Figure S1. Germinal center and antigen-specific CD4⁺ T cell responses induced by ORFV-S and Jcovden in homo- and heterologous vaccination regimens.** CD-1 mice were immunized on days 0 and 21 with 10⁶ PFU of ORFV-S, 1/10 of the human dose of Jcovden, or PBS. **A)** Number of germinal center (GC) B cells and T follicular helper (Tfh) cells per spleen on day 28. **B)** Number of spike-specific CD4⁺ T cells per spleen assessed on day 28 by intracellular cytokine staining (ICS) following *ex vivo* peptide restimulation. Heights of bars indicate mean ± SEM (standard error of the mean). **C)** Correlation between the numbers of antigen-specific CD4⁺ T cells per spleen and the corresponding total IgG endpoint titer in serum on day 28.


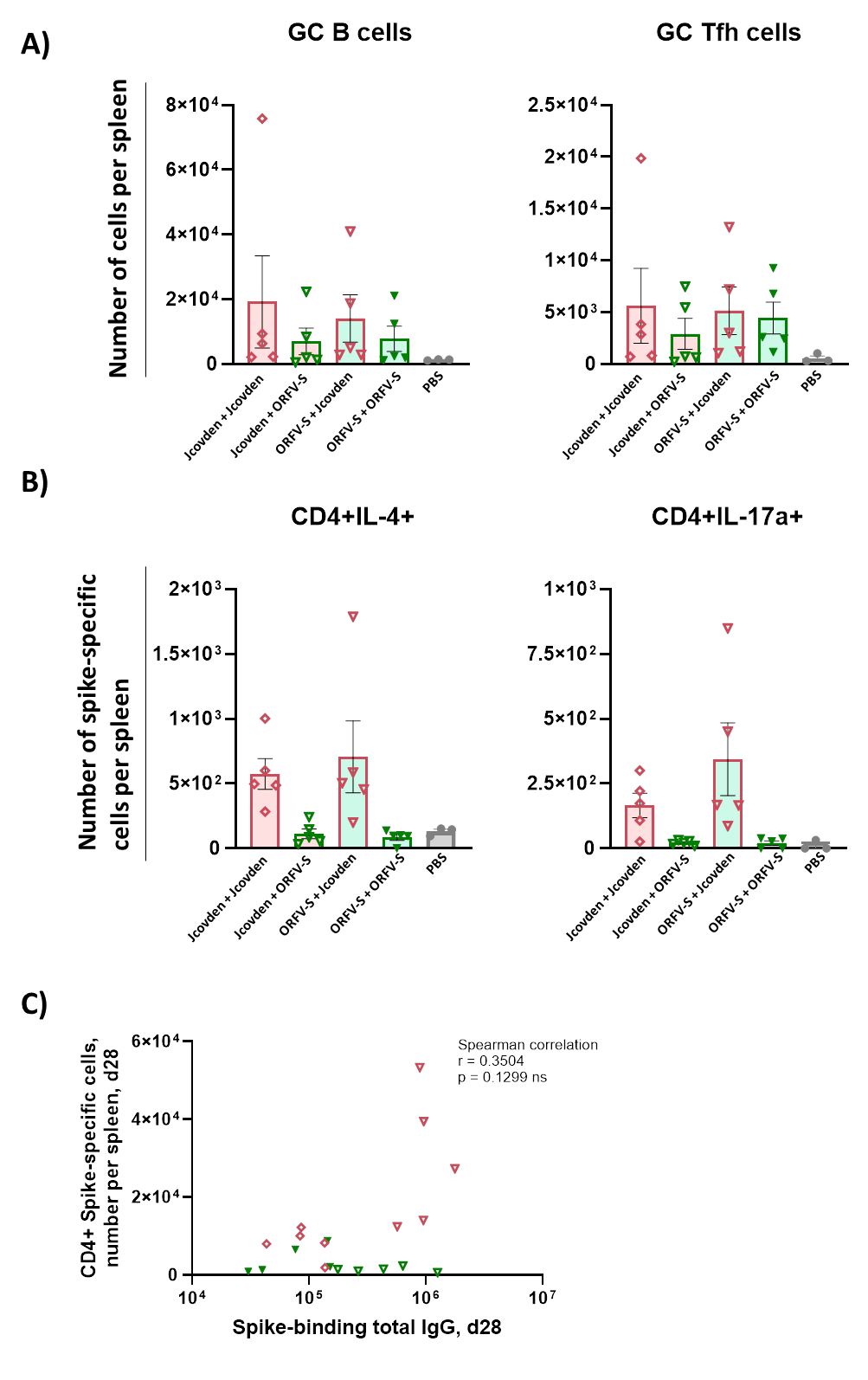

Supplement: Supplementary Figure 1 — Germinal center and antigen-specific CD4+ T cell responses induced by ORFV-S and Jcovden in homo- and heterologous vaccination regimens. CD-1 mice were immunized on days 0 and 21 with 106 PFU of ORFV-S, 1/10 of the human dose of Jcovden, or PBS. (A) Number of germinal center (GC) B cells and T follicular helper (Tfh) cells per spleen on day 28. (B) Number of spike-specific CD4+ T cells per spleen assessed on day 28 by intracellular cytokine staining (ICS) following ex vivo peptide restimulation. Heights of bars indicate mean ± SEM (standard error of the mean). (C) Correlation between the numbers of antigen-specific CD4+ T cells per spleen and the corresponding total IgG endpoint titer in serum on day 28. [file DataSheet1.docx]
